# Referral from primary care to a physical activity programme: establishing long-term adherence? A randomized controlled trial. Rationale and study design

**DOI:** 10.1186/1471-2458-9-31

**Published:** 2009-01-22

**Authors:** Maria Giné-Garriga, Carme Martin, Carlos Martín, Anna Puig-Ribera, Juan José Antón, Agustí Guiu, Ana Cascos, Rafel Ramos

**Affiliations:** 1 Department of Physical Activity and Health, Primary Health Care of Barcelona, Institut Català de la Salut. Barcelona, Spain; 2 Department of Physical Activity and Sport Sciences, FPCEE Blanquerna, Universitat Ramon Llull. Barcelona, Spain; 3 Research Unit of Barcelona, Primary Healthcare Research Institution IDIAP Jordi Gol. Barcelona, Spain; 4 Primary healthcare centre Passeig Sant Joan, Institut Català de la Salut. Barcelona, Spain; 5 Department of Medicine, Universitat Autònoma de Barcelona. Barcelona, Spain; 6 Department of Physical Activity and Sport Sciences, Universitat de Vic. Catalonia, Spain; 7 Primary healthcare centre Manso, Institut Català de la Salut. Barcelona, Spain; 8 Primary healthcare centre Serraparera, Institut Català de la Salut. Cerdanyola – Ripollet, Spain; 9 Research Unit of Girona, Primary Healthcare Research Institution IDIAP Jordi Gol. Girona, Spain

## Abstract

**Background:**

Declining physical activity is associated with a rising burden of global disease. There is little evidence about effective ways to increase adherence to physical activity. Therefore, interventions are needed that produce sustained increases in adherence to physical activity and are cost-effective. The purpose is to assess the effectiveness of a primary care physical activity intervention in increasing adherence to physical activity in the general population seen in primary care.

**Method and design:**

Randomized controlled trial with systematic random sampling. A total of 424 subjects of both sexes will participate; all will be over the age of 18 with a low level of physical activity (according to the *International Physical Activity Questionnaire*, IPAQ), self-employed and from 9 Primary Healthcare Centres (PHC). They will volunteer to participate in a physical activity programme during 3 months (24 sessions; 2 sessions a week, 60 minutes per session).

Participants from each PHC will be randomly allocated to an intervention (IG) and control group (CG). The following parameters will be assessed pre and post intervention in both groups: (1) health-related quality of life (SF-12), (2) physical activity stage of change (Prochaska's stages of change), (3) level of physical activity (IPAQ-short version), (4) change in perception of health (vignettes from the *Cooperative World Organization of National Colleges, Academies, and Academic Associations of Family Physicians*, COOP/WONCA), (5) level of social support for the physical activity practice (*Social Support for Physical Activity Scale*, SSPAS), and (6) control based on analysis (HDL, LDL and glycated haemoglobin).

Participants' frequency of visits to the PHC will be registered over the six months before and after the programme. There will be a follow up in a face to face interview three, six and twelve months after the programme, with the reduced version of IPAQ, SF-12, SSPAS, and Prochaska's stages.

**Discussion:**

The pilot study showed the effectiveness of an enhanced low-cost, evidence-based intervention in increased physical activity and improved social support. If successful in demonstrating long-term improvements, this randomised controlled trial will be the first sustainable physical activity intervention based in primary care in our country to demonstrate long-term adherence to physical activity.

**Trial Registration:**

A service of the U.S. National Institutes of Health. Developed by the National Library of Medicine. ClinicalTrials.gov ID: NCT00714831.

## Background

People who lead a physically active life or are in good physical shape have a lower mortality rate and a longer life expectancy [1,2]. The regular practice of physical activity (PA) has a positive effect in reducing obesity and preventing cardiovascular pathologies [3,4], reducing the risk of stroke [5], reducing the deterioration of the pulmonary function and the risk of suffering EPOX [6], prevents diabetes [7], increases HDL cholesterol and decreases triglycerides and total cholesterol [8]. It is also important in preventing falls in the elderly [9], generates a sensation of well-being, reduces anxiety and symptoms of depression, increases self-esteem [10], and improves the perception of quality of life related to health [11,12]. The World Health Organisation (WHO) has published a report in which physical inactivity is cited as one of the principle risk factors in the development of chronic illness and cause of death, especially in industrialised countries [13]. Even so, only 18% of the European population claims to practice moderate physical activity on a daily basis [14]. According to the National Health Survey of 2001, 46.6% of the Spanish population over the age of 15 does not exercise in their free time and only 8.5% exercise on a regular basis [15]. Furthermore, data from the Catalan Health Survey of 2006 indicate that less than 48% of the Catalan population exercises sufficiently to improve their health, a percentage which has increased since the surveys of 1994 and 2002 [16]. The virtual absence of a public health practice infrastructure for the promotion of PA at a local level presents a critical challenge to control policy for chronic disease, particularly obesity. Translating the increasing evidence of the value of PA into practice will require systemic, multilevel, and multisectorial intervention approaches that build individual capability and organisational capacity for behaviour change, create new social norms, and promote policy and environmental changes that support higher levels of energy expenditure across the population [17].

In 2005, the Spanish Ministry for Health and Consumption designed the Strategy for Nutrition, Physical Activity and Prevention of Obesity (NAOS) with the aim of promoting a healthy lifestyle [18]. To the same end, in Catalonia, the Department of Health of the Catalan Government launched an integrated plan for the Promotion of Health through Physical Activity and Healthy Diet (PAAS) [19].

Within this plan, encouraging PA promotion in PHC is outlined as a priority [19], as in Spain the high proportion of inactive primary care patients (at least 70%) justifies the need to develop a targeted strategy for physical activity promotion in general practices [20]. There is contradictory evidence about the effectiveness of including the usual advice on the practice of regular physical activity in the consulting rooms of PHC [21-23], and it is not certain if it is even applicable in our country [24].

In Catalonia, Puig-Ribera, McKenna and Riddoch (2005) indicated that medical recommendations for physical activity were limited, basically due to lack of time, unfavourable working conditions in healthcare centres and lack of knowledge on the part of the healthcare professionals, making it clear that the task of promoting physical activity was not seen as effective; concluding with the importance of establishing working protocols for consultants to integrate the promotion of physical activity in their work on a daily basis in the clinical practice [25].

Several types of intervention for the promotion of PA in Primary Care have been reported. It has been demonstrated that those which combine written instructions, an exercise programme and strategies to change behaviour, and which are accompanied by several training sessions, are more effective [26]. Thus it appears that the highest success rate is seen in those interventions which are not limited to professional advice given at the PHC [26,27]. In the same way a systematic review concluded that advice in routine primary care consultations was not an effective strategy means of producing sustained increases in physical activity [28].

Various authors have indicated the importance of referring patients to professionals specialised in the design of healthy exercise programmes outside the healthcare environment, making use of the local resources available in each area as a strategy for effective integration of the promotion of exercise in Primary Care [17,29]. In this context, a recent systematic review [30] assessed whether exercise-referral schemes were effective in improving exercise participation in sedentary adults. These schemes showed a small effect on increasing physical activity in sedentary people, partly due to poor rates of uptake and adherence to the exercise schemes [30]. Therefore, further studies are required to find strategies to increase long-term adherence [31] by addressing the participation barriers such as lack of social support, intimidating environments to practice a regular physical activity and inadequate supervision [30].

A systematic review of interventions based on the promotion of walking found only two studies in which there was a significant increase in the time spent walking and which improved the clinical risk indicators [32]. Finally, a Cochrane review (2005) showed moderate but positive utility regarding the interventions based on self informed physical activity, such as the effect on the cardio respiratory state [33]. The effect of the interventions in achieving a predetermined threshold of physical activity was not significant with an odds ratio of 1.30 (Confidence interval 95%: 0.87 to 1.95) [33]. In better quality studies, exercise was self directed with some professional guidance and with constant professional support [29].

In 2006, Giné-Garriga and Martin developed a pilot study in Barcelona (with a control group) of the Programme for the Promotion of Physical Activity in the PHC in which they offered patients the possibility of participating for a three month period in an exercise programme carried out in their own healthcare centre [34]. Patients were recruited in the healthcare centres, and physical activity specialists designed programmes which were specifically aimed at the patients' needs [34,35]. The sessions were carried out together with nurses and physical therapists from the centre. The programme was carried out in the centre itself, making use of nearby outdoor public spaces in order to offer the patients convenient and familiar surroundings. During the last sessions of the programme all the participants were given information about the nearest municipal facilities and the activities offered, and a visit to these facilities was organized. Thus, the programme acted as a means for incorporating participants in local facilities once they had experienced the benefits of doing regular exercise and at the same time used behavioural strategies [36], accompanied by people from the same neighbourhood with similar needs [34].

In order to achieve this objective, establishing a link between the PHC and the central offices of the various municipal districts was considered essential, as was contact with local sports centres, civic centres and other health centres, in order to facilitate the incorporation of the patient in a programme or exercise session, either individual or group, outside the environment of the healthcare centre. Facilitating access to the existing local resources in the area would contribute considerably to the continuity of the initiatives and programmes being developed by Primary Care. Furthermore, it would encourage a greater number of citizens to establish a common protocol of action [28].

## Method and design

### Aim

This randomised controlled trial was designed to assess the effectiveness of a primary care physical activity intervention, which considered community strategies coordinated with municipal resources and the incorporation of various professional disciplines, in increasing adherence to physical activity in the general population seen in primary care.

### Study population

Inclusion criteria will include (a) adult patients (over the age of 18) of both sexes who will be seen at the PHC for whatever reason; (b) with a low level of physical activity as established by the shortened version of the questionnaire *International Physical Activity Questionnaire *(IPAQ) [37]; (c) with a diagnosed chronic pathology; (d) who will be willing to participate in an exercise programme; and (e) who will have minimum physical aptitudes to follow the programme (being able to walk and stand up from a chair independently).

Exclusion criteria will be based on certain medical conditions which could result in unwanted effects in older adults, such as the presence of unstable angina, uncontrolled congestive heart failure, unstable arrhythmia or heart valve disease, progressive or debilitating medical conditions, and severe hypertension (systolic ≥ 200, or diastolic ≥ 120) [38].

Written informed consent will be obtained from all subjects of both intervention (IG) and control group (CG). This trial was approved by the Clinical Investigation Ethics Committee of the IDIAP Jordi Gol, located in Barcelona.

### Recruitment process

**Figure 1:**
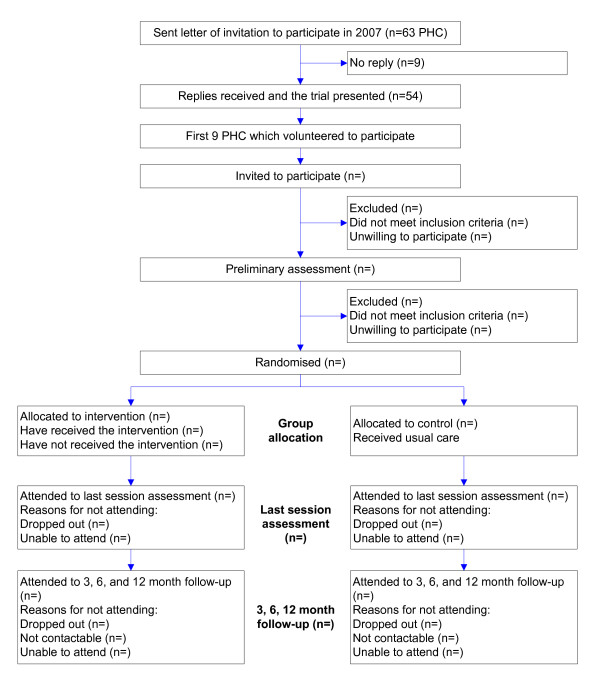
**Flowchart of participant's recruitment and trial design**.

Recruitment will take place in 9 PHC in different regions of Catalonia during the first three months of 2009. Until August 2008, 63 PHC in Catalonia were informed and the trial has been presented to the 54 centres which showed interest in participating. Of these, the first nine centres which volunteered to participate will undergo the trial. Two health professionals, who were selected on a voluntary basis from each of the participating centres, were trained. During the recruitment period, the opportunity to participate in the study will be offered daily to all patients with a chronic pathology, who by systematic random sampling will be previously identified in the doctors' lists: a total of 50 subjects per centre will be recruited. The patients who meet the inclusion criteria and agree to participate will be contacted to inform them about how the project will be carried out. A record of demographic and health data, as well as attendance at the sessions, will be kept of those who agree to participate in the intervention. Figure 1 shows the flowchart of participants' recruitment and trial design.

### Randomisation

Previously trained professionals in each PHC will invite the subjects to participate in the study, following random systematic procedures during the recruitment period. All the subjects who meet the inclusion criteria must sign a consent form, carry out an initial evaluation and following this they will be randomly selected to form part of the IG or the CG. Random sequence generation will be computer-generated by an independent researcher, guaranteeing that the allocation groups remain confidential.

### Control group

Subjects assigned to the control group will be asked to continue their routine daily activities and will receive their usual care from their primary care practice. They will be given information and requested to give their informed consent. They will be invited to the first and the last session (3 months later) of the programme in which the different parameters will be evaluated. They will be reminded by telephone two months and three weeks prior post-intervention assessment session.

### Intervention

#### Physical activity promotion programme

When a patient agrees to participate an assigned professional health worker will send a registration form to the investigation team and to the specialist who will lead the exercise group. The patient will be contacted by telephone to carry out the evaluation and to start the intervention. All participants will report to the healthcare centre twice a week for 12 weeks, and all participants who hand in a consent form will undergo an analysis stating their intention to undergo the treatment.

Each session will last 60 minutes, and all protocols have been developed for progressive intensity. The sessions will be carried out by physical activity specialists in a room in the health centre itself, in nearby exterior locations (parks, squares, etc) or in municipal facilities in the same district. Table 1 shows the contents of the programme [35]. At the same time, participants will be shown exercises that they should do at home. It is recommended that from the first day, a minimum of three sessions per week be done at home without supervision and without keeping a record.

**Table 1:** Components of the exercise programme [35]

**Component**	**Includes these exercises**	**Repetitions per exercise**^a^	**Duration of a single exercise or repetition**	**Duration per 60 minutes class**	**Major benefits**
**Warm-up**	- Range-of-motion. - Low-intensity aerobics.	4–8	2 seconds per repetition	10 minutes minimum	- Increases internal body temperature. - Injury prevention.

**Aerobics**	- Aerobic exercises (e.g. walking, swimming, cycling)	Varies	Varies	15–30 minutes	- Cardio respiratory endurance.

**Resistance training**	- Body- weight^b ^exercises. - Resistance exercises.	8–15	6 seconds per repetition	15–30 minutes	- Muscular strength and endurance.

**Cool-down**	- Stretching.	1	30–45 seconds per stretch	5–30 minutes	- Improved flexibility.

	- Relaxation techniques. - Stress-reduction techniques.	Varies	Varies	5–30 minutes	- Relaxation, stress reduction.

^a^Several factors determine the number of repetitions, such as the component of exercise, the level of fitness of the participant, the level of progress for that exercise, day-to-day variations in the participant's state, and the total amount of time available for the exercise class.

^b^Body-weight exercises are exercises in which one's body weight, such as the weight of one's arm or legs, serves as the resistance.

To facilitate continuity in the carrying out of physical exercise once the programme has finished, all participants will be offered a personalised exercised programme with exercises that have been performed during the sessions so that the subject can continue the activity as a matter of habit. Moreover, a list of local resources (sports facilities, civic centres, etc.) in the same district where the activity can be continued on a regular basis will be given. At the same time the penultimate session of the intervention will be carried out in a nearby sports facility with a previously programmed visit. During the first and last sessions, a baseline and final evaluation of the variables to be studied will be carried out.

### Blinding – single blind

Baseline measures will be taken prior to allocation of randomisation. Independent investigators assessing participants at the end of the programme and at three, six and 12 month follow-up visits or phone calls will be blind to the allocation of the treatment group. Participants will be asked not to discuss group allocation with the assessing professional. Moreover, the person who carries out the analysis of the data will not be involved in the investigation.

### Outcome measures

#### Main objective

The primary outcome measured is adherence, means of increasing physical activity level assessed with short-version IPAQ questionnaire [37]. The study measure will be assessed at baseline, at the end of the intervention, and at three, six and twelve months follow-up.

The level of physical activity will be evaluated, moving from a low level to a moderate or high level, or increasing the weekly number of Metabolic Equivilent Units (MET). The IPAQ enables physical activity to be evaluated as a continuous variable by calculating the weekly METs, and classifying the subjects according to whether they have a low, moderate or high level of physical activity. This instrument has shown good validity and reliability in general population in a previous study [37].

#### Other specific objectives

Secondary outcomes for IG and CG include: (a) decreasing the number of visits to the healthcare centre by individuals involved in the programme, registering the total number of visits during the six months before and after the programme; (b) description of the development of quality of life related to health, measured with SF-12 test [39,40] (the first and last session of the programme) and evaluation of short, medium and long term evolutions, with follow up at three, six and twelve months after the programme has finished; c) description of the evolution of perceived health status, measured with the COOP/WONCA vignettes (first and last session of the programme) [41]. This instrument showed good validity and reliability in general population in a previous study [41]; (d) evaluation of the attitude towards the change in behaviours in relation to regular exercise using the Prochaska scale (first session and six and twelve months after finalising the project) [42]; (e) evaluation of the social support for the physical activity practice, assessed using the SSPAS scale [43] (first and last session of the programme and three, six and twelve months after finalising the intervention). Reliability and validity of the scale was previously assessed in an elderly population [43]; and (f) description of the evolution of HDL, LDL and glycosilate hemoglobin (first and last session and 6 and 12 months after finalising the programme). Demographic and health data will be also collected (age, blood pressure, heart beat when resting, weight, height, body mass index, current medication and associated pathologies).

### Data management and quality assurance

Data will be entered directly into a customized Microsoft Access database by investigators at the time of the interview and baseline testing. Daily backups will be performed and transferred to the master database at least once a week. Random checks of data entry will be performed regularly and corrections made will be possible by checking against paper records or, in rare cases, by phoning participants for confirmation by independent investigators.

### Sample size

Accepting an alpha risk of 0.05 and a beta risk of 0.20 in a bilateral contrast, 424 individuals are needed: 212 individuals in the IG and 212 in the CG in order to detect a difference equal to or higher than 0.15 between the two [23]. A proportion of 0.5 in one of the groups is to be assumed. A dropout rate of 16% is estimated, based on the pilot study experience [34].

### Statistical methods

Analysis of effectiveness will be made followed by the intention to treat analysis. The analysis of data will be made using the SPSS statistic software version 15.0 and STATA, version 9.1. In all cases a bilateral alfa error of 0.05 will be used and the confidence intervals will be calculated at 95%. Descriptive statistics will be calculated and intervention and control groups will be checked for health and outcome measures at baseline.

A baseline comparability analysis of the CG and IG in relation to the variables studied, will be carried out. An s-Student test or ANOVA will be used in the comparison of means if the variables follow a normal distribution and the U of Mann Whitney if they do not. For the other dimensions of the analysis, a covariance analysis (ANCOVA) for repeated measures will be carried out.

For the multi-variance analysis the change in the time of the described dependent variables will be evaluated, and comparisons between the CG and IG will be established. To do this multilevel linear models will be adjusted, one for each dependent variable. In the first level the individual path or the evolution of each individual over a long period of time (pre-post intervention, 3,6,12 month follow-up) will be modelled. In the second level it will be adjusted according to the variables that refer to the individual; the intervention variable will be added (intervention or control) to the independent variables described earlier.

### Cost-effectiveness analysis

This data will be used in a subsequent cost effectiveness analysis in terms of welfare pressure, should the trial prove positive.

### Ethical approval

This trial was approved by the Clinical Investigation Ethics Committee of the IDIAP Jordi Gol, located in Barcelona. The participation of the subjects is strictly voluntary and withdrawal will not have any consequence on the management of their illness which will be carried out by their doctor strictly following the accepted international norms. The data will be treated with utmost confidentiality according to the Organic Law which regulates the confidentiality of computerised data (Protection of personal information Law 15/1999), and will be used exclusively for the purposes of this scientific investigation.

## Discussion

Primary healthcare is an ideal setting to identify adults who are physically inactive and to initiate a brief, cost-effective physical activity intervention. As is indicated by the National Institute for Health and Clinical Excellence (NICE) Clinical Guideline, local strategies which coordinate municipal resources with PHC are needed in order to promote physical exercise, especially in vulnerable groups. At the same time, the incorporation of professionals from different disciplines should be considered in order to increase the practice of long term physical exercise (grade of evidence C) [44].

It should be considered that the evidence is still insufficient in key areas and it is necessary to broaden the investigation in the following: (a) evaluating whether an intervention based on practical exercise sessions along with written material provided by PHC is more effective than the usual advice given, in increasing levels of physical activity in the short, medium and long term, as well as evaluating the attitude of the population towards regular exercise; (b) evaluating whether an intervention which includes community strategies which coordinate municipal resources with those of the primary healthcare centre, and also consider involving professionals from various disciplines, is effective in increasing the levels of physical exercise in the general population; and (c) evaluating the effect that current intervention with social support has in the short, medium and long term in maintaining physical exercise, using the Social Support for Physical Activity Scale (SSPAS) [43].

High demand is one of the current problems facing primary healthcare in our country. In a study carried out in Sweden, it was concluded that encouraging exercise from primary healthcare is cost effective in terms of reducing the utilisation of healthcare resources, the number of admissions to hospital and visits to healthcare centres, especially when the intervention is addressed to elderly people with more than one cardio-vascular risk factor (sedentary lifestyle, hypertension, diabetes, obesity, among others) [45].

A recent systematic review of exercise-referral schemes showed only a small effect on increasing physical activity in sedentary people partly due to poor rates of uptake and adherence to the exercise schemes [31,32]. The design of the current study (PPAF) uses the advantage of direct contact and accessibility to primary healthcare with patients. Twenty-four sessions are carried out by a specialist in physical exercise in the same primary healthcare centre where the individuals are listed, taking advantage of patient familiarity with the space, and in conjunction with a group of people from the same neighbourhood and with similar characteristics. Upon finalising the intervention, all participants will be offered access to the most appropriate resources in the district after a programmed appointment.

To ensure the quality of this randomised clinical trial the guide developed by the CONSORT statement (Consolidated Standards of Reporting Trials) [46] has been followed.

## Competing interests

The authors declare that they have no competing interests.

## Authors' contributions

All authors contributed to the study design and development of the trial protocol. MGG and CMB are the principal investigators of the study, and CM and AP the trial managers. AG, JJA, RR and AC are co-investigators. MGG and CMB drafted the paper and CM, AP and RR revised the manuscript critically and contributed to subsequent drafts. All authors read and approved the final manuscript.

## Pre-publication history

The pre-publication history for this paper can be accessed here:


